# David Herbert Llewellyn

**Published:** 1864-09

**Authors:** 


### OBITUARY.

### *The Surgeon of the Alabama.*

David Herbert Llewellyn, who perished in the noble performance of his duty in the late action off Cherbourg, was the son of the Rev. David Llewellyn, perpetual curate of Euston Royal, Wilts. He was educated at Marlborough College, was an articled pupil of Dr. Hassall, of Richmond, and subsequently studied his profession at Charing-Cross Hospital, from 1856 to 1859. He was Silver Medalist in surgery and chemistry. Of a generous and ardent disposition, he gained the esteem and regard of all his fellow-students. He was with the Alabama throughout the whole of her eventful career, end was much respected by all on board. We are enabled to give a copy of the last letter which we believe he ever wrote. It was addressed to Mr. Travers, the resident medical officer of Charing-Cross Hospital, and is as follows:

CHERBOURG, June 14th, 1864.

"DEAR TRAVERS.—Here we are. I send this by a gentleman coming to London. An enemy is outside. If she only stays long enough, we go out and fight her. If I live, expect to see me in London shortly. If I die, give my best love to all who know me. If Monsieur A. de Caillet should call on you, please show him every attention." I remain, dear Travers, ever yours,

D. H. LLEWELLYN.

How poor Llewellyn did his duty as a man and a surgeon may be judged by the following touching episode which was seen to occur during the late battle: "The whaleboat and dingy, the only two boats uninjured, were lowered, and the wounded men placed in them, Mr. Fulham being sent in charge of them to the Kearsage. When the boats were full, a man who was unwounded endeavored to enter one, but was held back by the surgeon of the ship, Mr. Llewellyn. 'See,' he said, 'I want to save my life as much as you do; but let the wounded men be saved first.' 'Doctor,' said the officer in the boat, 'we can make room for you.' 'I will not peril the wounded men,' was his reply." He remained behind, and sank with the ship—a loss much deplored by all the officers and men.

Noble and self-denying as was the conduct of the late surgeon of the Alabama, we are proud in the conviction that the same chivalrous spirit animates the medical officers of the united services of this kingdom. There has been much talk of their being "non-combatant officers;" but where are we to look for greater heroism or self-devotion ' even at the cannon's mouth." And yet Llewellyn was the type of a class whom the Admiralty and the Horse Guards have thought fit, by every means in their power, to degrade and insult. No wonder, under such circumstances, that the service is now so unpopular that there are more than 200 vacancies which cannot be filled up. The cause in which the real hero of the late naval duel perished is not one which can be acknowledged by any national testimonial; but we are glad to hear that his fellow-students contemplate the erection of a tablet to his memory in the hospital in which he so greatly distinguished himself, and in which his kindly and generous spirit had gained for him the greatest esteem and affection. It would be a fitting monument to his memory, and we trust that it will be placed in so appropriate a position.—*Lancet*, Aug, 1864.

